# Dysfunction of the frontolimbic region during swear word processing in young adolescents with Internet gaming disorder

**DOI:** 10.1038/tp.2015.106

**Published:** 2015-08-25

**Authors:** J-W Chun, J Choi, H Cho, S-K Lee, D J Kim

**Affiliations:** 1 Department of Psychiatry, Seoul St Mary's Hospital, The Catholic University of Korea College of Medicine, Seoul, Korea; 2 Department of Psychiatry, Hallym University College of Medicine, Chuncheon Sacred Heart Hospital, Chuncheon, Korea

## Abstract

Although the Internet is an important tool in our daily life, the control of Internet use is necessary to address difficult problems. This study set out with the aim of assessing the cognitive control of affective events in Internet gaming disorder (IGD) and has examined the influence of IGD on neural activities with regard to swear words in young adolescents. We demonstrated the differences between adolescents with IGD and healthy control adolescents (HC) with respect to swear, negative and neutral word conditions. Swear words induced more activation in regions related to social interaction and emotional processing such as the superior temporal sulcus, right temporoparietal junction and orbitofrontal cortex (OFC) when compared with negative words. In this study, adolescents with IGD exhibited reduced activation in the right OFC related to cognitive control and in the dorsal anterior cingulate cortex (dACC) related to social rejection during the swear word condition. In addition, adolescents with IGD were negatively correlated with activity in the right amygdala toward swear words, indicating the important role of the amygdala in the control of aggression in adolescents with IGD. These findings enhance our understanding of social–emotional perception in adolescents with IGD.

## Introduction

The past two decades have seen increasingly rapid advances in the Internet as a medium for activities that we use to make our daily life convenient and that we regard as important parts of our life such as banking, purchasing movie tickets, making reservations, reading news and a host of others. However, the number of people experiencing negative effects from excessive Internet use, such as loss of control over their Internet use and social problems including school and/or work difficulties, has also grown extensively with the growth of the Internet.^1, 2^ In previous studies, Internet addiction and pathological Internet use have been defined as compulsive and excessive Internet use, featuring withdrawal symptoms, increased tolerance and negative repercussions including social isolation and poor academic or professional achievement.^3, 4^

Using data from 2012, the South Korean government estimated that ~754 000 South Korean adolescents (10.7% ages 10–19) were afflicted and required treatment and that the Internet addiction of adolescence is more serious than that of any other age range.^5^ It was also determined that 78% of adolescents use Internet games. Despite the growing concern about problematic Internet/Internet gaming use, a consensus about the diagnosis and assessment of the relevant disorder has not yet been reached among researchers and clinicians. Internet gaming disorder (IGD) has been included in Section 3 of the research appendix of the Diagnostic and Statistical Manual version-5 (ref. 6) and is an issue in the field of behavioral addiction. IGD, a subtype of Internet addiction,^4^ is related to the compulsive use of online games. In previous studies, it has been shown that the principal behavior criterion of IGD is the loss of control over Internet use and is represented as persistence in online gaming use despite the awareness that it is directly harmful to one's psychosocial performance.^7, 8, 9^

Internet addiction is particularly harmful to brain development in adolescence. Adolescence is a time of considerable development in behavior, cognition and the brain, and, thus, it seems to be much more difficult to coordinate executive functions and social cognitive abilities within brain networks after puberty.^10^ With respect to executive function, adolescents with IGD have tendencies to be highly impulsive, lack problem-solving skills and be easily distracted in communicating with others.^11, 12^ In a previous study related to executive function, individuals with Internet addiction exerted more effort when confronted with complex situations of decision-making or when cognitive flexibility was necessary.^13^ The impairment of error monitoring in subjects with Internet addiction is related to stronger activity in the anterior cingulate cortex (ACC),^14^ and executive and decision-making functions may be even worse when Internet-related stimuli were presented.^1, 11^ Indeed, it has been reported that Internet-addicted adolescents showed lower gray matter density in the ACC and lower fractional anisotropy in the orbitofrontal white matter and cingulum compared with healthy control (HC).^15, 16^ In addition, male adolescents with Internet addiction have significantly decreased cortical thickness in the right lateral orbitofrontal cortex (OFC),^17^ a brain region that is involved in craving and compulsive repetitive behaviors that reflect shared behavioral tendencies in addiction and obsessive-compulsive disorder.^18, 19^ Therefore, dorsal anterior cingulate cortex (dACC) and OFC are considered to be the *a priori* regions related to cognitive control and executive function.

Adolescents with Internet addiction are also more likely to exhibit aggressive behavior,^20^ and aggression is positively correlated with online gaming addiction.^21, 22^ Various studies illustrate that adolescents who spend more time on the computer- or in Internet-mediated environments are more associated with cyberbullying^23^ and verbally aggressive behaviors such as insulting and swearing.^24, 25^ In South Korea, cyber violence in an Internet-mediated environment has become a social problem. Approximately 75% of adolescents 12–19 years of age reported experiencing cyber violence, and 87.6% of Internet users in elementary schools reported using swear words, one type of cyber violence, on the Internet.^26^ Thus, understanding how Internet gaming influences aggressive behavior in adolescents is important in the development and implementation of preventive strategies against adolescent cyber violence.^27^ In particular, investigations related to cyber violence such as the use of swear words are important in Internet-mediated environments.

Swear words, in particular, express strong emotion, mostly to reveal anger and frustration.^28^ Although swearing has adaptive functions such as a marker of group solidarity^29^ and increase of pain tolerance,^30, 31^ it has been reported that swear words are related to social threatening^32^ and are a strong physiological response induced by an affective impact.^33^ This paper will focus on cognitive control of the strong emotional responses induced by swearing on the neural activity. Therefore, the amygdala, whose activity is related to the strong affective response^34, 35^ and is correlated with cognitive control, was selected as an *a priori* region.

In summary, the aim of this study is to investigate (1) the neural activities during the processing of swear words that represent aggression and (2) the correlation between neural activities in response to swear words and cognitive control in young adolescents with IGD compared with HC. In this study, the frontolimbic regions including the dACC, OFC and amygdala were considered to be the *a priori* regions related to cognitive control in response to swear words: the dACC is involved in monitoring, the OFC in craving and compulsive repetitive behaviors and the amygdala in the affective response.

## Materials and methods

### Participants

This study focused on male adolescents because the prevalence of IGD is much higher in male than in female adolescents, and there might be sex differences related to swearing. A total of 716 male adolescents 12–15 years of age participated in the survey at two middle schools in Kangwon-do, South Korea. Nineteen adolescents with IGD and nineteen HC were recruited for the functional magnetic resonance imaging (fMRI) study. In addition, all participants underwent a structured interview based on the Korean Kiddie-Schedule for Affective Disorders and Schizophrenia (K-SADS-PL) by a clinician.^36^ Of the adolescents with IGD, three participants were excluded because of depressive disorder and attention deficit hyperactivity disorder, and, thus, the data of 16 adolescents with IGD (13.63±1.03 years) and 19 HC (13.37±0.90 years) were considered in this study (Table 1). Exclusion criteria included past or current major medical disorders (for example, diabetes mellitus), neurological disorders (for example, seizure disorders, head injury) or psychiatric disorders (for example, major depressive disorder, anxiety disorders). All participants had normal or corrected-to-normal vision and were right-handed (as assessed by the Edinburgh handedness inventory).^37^ The purpose and procedure of this study were explained to the participants and their parents. Each participant provided written informed consent, and this study was approved by the Institutional Review Board of Seoul St Mary's Hospital.

### Questionnaires

Internet addiction was estimated using the Korean Internet Addiction Proneness Scale (the K-scale) developed by the South Korean government in 2002. The K-scale is a self-report scale and includes 15 items that are scored on a four-point Likert scale (1: Not at all to 4: Always). The K-scale has six subscales: daily life disturbance, disturbance of reality testing, automatic addictive thoughts, virtual interpersonal relationships, deviant behavior and tolerance.^38^ The reliability and validity of the K-scale has been established for elementary school and middle and high school students.^38^ In addition, all participants completed the Conners–Wells' Adolescent Self-Report Scale-Short Version (CASS-S) to assess Attention Deficit/Hyperactivity Disorder symptoms.^39^ The severity of depressive symptoms was assessed using the Beck Depression Inventory.^40^ All of the adolescents with IGD in this study were categorized as Internet addicted according to the K-scale, and the time of Internet game use was significantly higher than HC, even though the time for other Internet use excluding Internet gaming was comparable to HC.

All participants completed the Block Design and Vocabulary subtests of the Korean-Wechsler Intelligence Scale for Children, 4th edition (K-WISC-IV).^41^ To determine the cognitive control for swear words, we also considered the Anger Control subscale of the Korean State-Trait Anger Expression Inventory (STAXI-K).^42^ The STAXI-K is a 44-item self-reported questionnaire assessing anger-related traits, and the Anger Control subscale measures the ability of an individual to control angry feelings to avoid expressing anger. The Cronbach's alpha score of the Anger Control subscale of the STAXI-K is 0.88.^43^

### Experimental paradigm

The stimuli consisted of neutral words extracted from the modern Korean vocabulary frequency list^44^ (for example, tree (*namu*), desk (*chaecksang*), pencil (*yeonpeal*)), negative emotional words selected from the Korean affective word list^45^ (for example, murder (*salin*), suicide (*jasal*), filthy (*ohmul*)) and swear words extracted from the Korean abusive language survey for adolescents^46^ (for example, fuck (*ssibal*), crazy bitch (*michinnuen*), asshole *(gaesaekki*)). The neutral word stimuli were used to control the potentially confounding effects of linguistic characteristics, and the negative emotional word stimuli were considered as an experimental condition to investigate the effect of unpleasant emotions compared with swear word stimuli. The stimulus syllables consisted of more than two and fewer than four. On each trial, a single word was presented in the center of the screen. Participants were asked to discriminate the level of the negative feeling induced by the word that was randomly presented among three categorized words using three buttons while undergoing fMRI scans (1: Not at all negative, 2: Somewhat negative and 3: Extremely negative). The task sequences were composed of a rapid event-related design, in which the duration of each trial was 2500 ms and the interval between trials was jittered from 500 to 4500 ms using the Optseq2 program (http://surfer.nmr.mgh.harvard.edu/optseq/). The word stimulus lasted for 1800 ms, and a crosshair followed for 700 ms in each trial. The session started with dummy scans for 5 s, followed by 120 events consisting of 40 swear, 40 negative and 40 neutral word trials, and, thus, the trial took a total duration of 8 min 45 s.

### Image acquisition

Functional and structural MRI data were acquired using a 3T MRI system (Siemens, MAGNETOM Verio, Erlangen, Germany) equipped with an 8-channel head coil. Participants' heads were cushioned with attached earmuffs. The functional images were obtained using a T2*-weighted gradient echo-planar imaging sequence (38 slices, 4 mm thickness and no gaps, repetition time=2500 ms, echo time=30 ms, flip angle=90°, image matrix=64x64, field of view=220 mm and voxel resolution of 3.75 × 3.75 × 3.85 mm). Structural images with a resolution of 0.5 × 0.5 × 1 mm were acquired using a three-dimensional T1-weighted gradient echo sequence (1 mm thickness, repetition time =1780 ms, echo time=2.19 ms, flip angle=9°, image matrix=512 × 512 and field of view=240 mm).

### Data analysis

#### Behavioral data

Behavioral data were analyzed according to the emotional words (swear, negative and neutral words) and groups (adolescents with IGD and HC). Negativity discrimination and reaction time (RT) were measured and then analyzed by repeated measures analysis of variance to assess the main effects and interactions using IBM SPSS Statistics for Windows, Version 20.0 (IBM SPSS, Armonk, NY, USA). Subsequent paired *t*-tests for *post hoc* analyses were performed to test the significance between different conditions and groups. All the levels of significance (alpha) for behavioral data were set to 0.05 after false discovery rate (FDR) control for multiple comparisons.

#### Image data

Image preprocessing and statistical analysis were performed with the Statistical Parametric Mapping software (SPM8; http://www.fil.ion.ucl.ac.uk/spm/software/spm8/; Wellcome Department of Cognitive Neurology, London, UK). The researchers who checked the quality of all images and performed the data-preprocessing procedure were blind to the sample identity. T1-weighted images were segmented into white matter, gray matter and cerebrospinal fluid using a skull-strip template image. After discarding the first two images from the dummy scan, the remaining 208 images were used for further processing. Differences in the slice acquisition time of the interleaved sequence were corrected, and realignment was performed to correct the errors caused by head motion. The corrected images were co-registered on the segmented T1-weighted image of the same participant. The co-registered T1 image was used as a source image in the normalization, and the corrected images were normalized to the standard T1 template. Functional data were smoothed with a Gaussian kernel of 8-mm full-width at half-maximum.

Preprocessed data were analyzed using a general linear model. Experimental trials were modeled separately using a canonical hemodynamic response function for individual data. Multiple linear regression, as implemented in SPM8 using a least-squares approach, was used to obtain the parameter estimates. These estimates were then analyzed by testing specific contrasts using the participant as a random factor. For the first-level analysis, we defined two conditions, SWEA (swear-neutral word condition) and NEGA (negative-neutral word condition). Images of the parameter estimates for each condition were created on the first-level analysis, during which individual realignment parameters were entered as regressors to control movement-related variance. In addition, we conducted a parametric modulation analysis by including the RT of each trial in the single subject level to remove the potentially confounding effect of movement processes.

For the second-level analysis, the parameters from each condition that were estimated in the first-level analysis were entered into a flexible factorial model, in which contrast maps for the main effects and interactions were analyzed. The results were measured using a 2 (emotional word: SWEA, NEGA) x 2 (group: adolescents with IGD, HC) design. The CASS-S and Beck Depression Inventory score were controlled in the second-level analysis using regressors. For comparison between conditions and groups, significant results were determined by FDR-corrected *P-*values of less than 0.05 and more than 50 voxels preferentially. Because we had four *a priori* regions, including the OFC, dACC and the bilateral amygdala related to cognitive control and affective response in swear words, we generated spherical regions of interest (ROIs) (radius=5 mm) centered on the peak of the Montreal Neurological Institute (MNI) coordinates in the activation map of the SWEA–NEGA condition: left amygdala (−20, −4, −18), right amygdala (34, 4, –20), dACC (0, 0, 34) and right OFC (52, 30, –6). The % BOLD signal changes in the ROIs were extracted in each condition using MarsBaR version 0.41 (http://marsbar.sourceforge.net) and were analyzed using repeated measures analysis of variance to investigate the differences between the groups and conditions under FDR-corrected *P*<0.05. The regional correlation was explored by calculating the correlations of the % BOLD signal changes between the ROIs using Pearson correlation analyses under the SWEA condition. When significant correlation (FDR-corrected *P*<0.05, two-tailed) was observed, moderation analyses were conducted to examine whether IGD affects the direction or magnitude of the relationship between two ROIs. Pearson correlation analysis was also performed to investigate the association between the Anger Control subscale of the STAXI-K and right amygdala activity in the SWEA–NEGA condition, and then, moderation analysis was used to determine the effects of Internet gaming addiction on this association.

## Results

### Demographic and clinical data

Table 1 summarizes the demographic and clinical characteristics of the two groups. The two groups did not differ in age, family monthly income, the Block Design and Vocabulary subtests of the K-WISC, and the score of the Anger Control subscale of the STAXI-K. Whereas the time for Internet excluding Internet game use per week was not different between groups, the time for Internet game use per week and the K-Scale score were significantly different.

### Behavioral data

Behavioral results are shown in Table 2. For negativity discrimination of words, the word conditions revealed main effects (F_2,66_=71.73, *P*=0.0001). The participants reported that swear (*t*=9.61, degrees of freedom (df)=34, *P*=0.0002) and negative words (*t*=9.75, df=34, *P* =0.0002) were more negative compared with neutral words. There was no significant difference between the two groups, and the interaction between words and groups was not significant.

For the RT, a significant difference was observed among the word conditions (F_2,66_=22.96, *P*=0.0001). The RT for negative words was delayed compared with that for swear (*t*=7.21, df=34, *P*=0.0002) and neutral words (*t*=5.02, df=34, *P*=0.0002). The interaction between words and groups for the RT revealed a significant difference (F_2,66_=3.78, *P*=0.03). The RT for negative words was slower compared with that for swear words in HC (*t*=10.02, df=18, *P*=0.0003), whereas the difference was not significant in the adolescents with IGD (*t*=2.67, df=15, *P*=0.06). In the group difference, HC showed a slower response than adolescents with IGD to negative words (*t*=2.04, df=33, *P*=0.049), and a delayed response to negative words compared with neutral words was exhibited only by HC (*t*=6.16, df=18, *P*=0.0001).

### Imaging data

#### Swear versus negative words

Results from the word condition analysis are presented in Table 3. In the SWEA condition, compared with the NEGA condition, the participants exhibited higher activity in the bilateral lingual gyrus, right superior temporal sulcus, right postcentral gyrus, bilateral orbitofrontal gyrus, right temporal pole, right temporoparietal junction, left precuneus and right rolandic operculum. The neural activity in the NEGA condition was not significantly different in the two groups compared with SWEA after FDR correction.

#### Group differences

Results of group comparisons are also presented in Table 3. In the SWEA condition, adolescents with IGD showed less activity in the left inferior frontal gyrus, left caudate nucleus and right middle temporal gyrus compared with HC. However, adolescents with IGD did not reveal significantly more activity than HC in the SWEA condition. In the NEGA condition, adolescents with IGD exhibited stronger activation in the right superior temporal gyrus compared with HC.

#### ROI analysis

**Figure 1:**
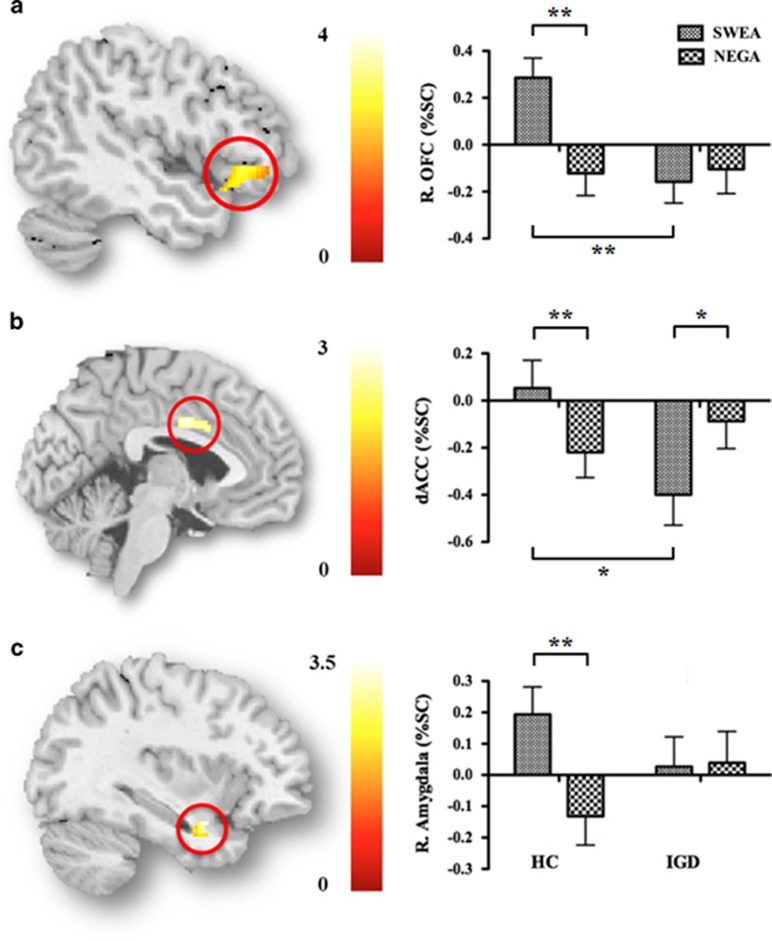
Brain activity of each region of interest (ROI) in the swearing-neutral (SWEA) condition. (**a**) Right orbitofrontal cortex (OFC; *x*, *y, z*=52, 30, −6), (**b**) dorsal anterior cingulate cortex (dACC; *x*, *y*, *z*=0, 0, 34), (**c**) Right amygdala (*x*, *y*, *z*=34, 4, −20). **P<0.005, *P<0.05.

In terms of the activity in the left and right amygdala and right OFC, the main effects of word conditions were significant (F_1,33_=15.65, *P*=0.0004; F_1,33_=7.21, *P*=0.015; F_1,33_=7.26, *P*=0.015, respectively), and the activity of left and right amygdala and right OFC were higher in the SWEA condition than in the NEGA condition (*t*=4.06, df=34, *P*=0.0004; *t*=2.67, df=34, *P*=0.019; *t*=2.60, df=34, *P*=0.019, respectively). As shown in Figure 1, there were interactions between word condition and group in the right amygdala, dACC and right OFC (F_1,33_=8.46, *P*=0.008; F_1,33_=19.95, *P*=0.0004; F_1,33_=12.46, *P*=0.002, respectively). In the right OFC, HC showed greater activity in the SWEA than in the NEGA condition (*t*=5.10, df=18, *P*=0.0004), but adolescents with IGD did not show a significant difference. In the dACC, HC showed significantly greater activity in the SWEA than in the NEGA condition (*t*=3.42, df=18, *P*=0.003), but adolescents with IGD showed stronger activity in the NEGA than in the SWEA condition (*t*=2.92, df=18, *P*=0.044). In the right amygdala, HC showed greater activity in the SWEA than in the NEGA condition (*t*=3.71, df=18, *P*=0.003), but adolescents with IGD did not show a significant difference. In particular, HC compared with adolescents with IGD showed significantly more activity in the dACC and right OFC in the SWEA condition (*t*=2.59, df=18, *P*=0.028; *t*=3.58, df=18, *P*=0.004). There were no significant group differences in the NEGA condition.

**Figure 2:**
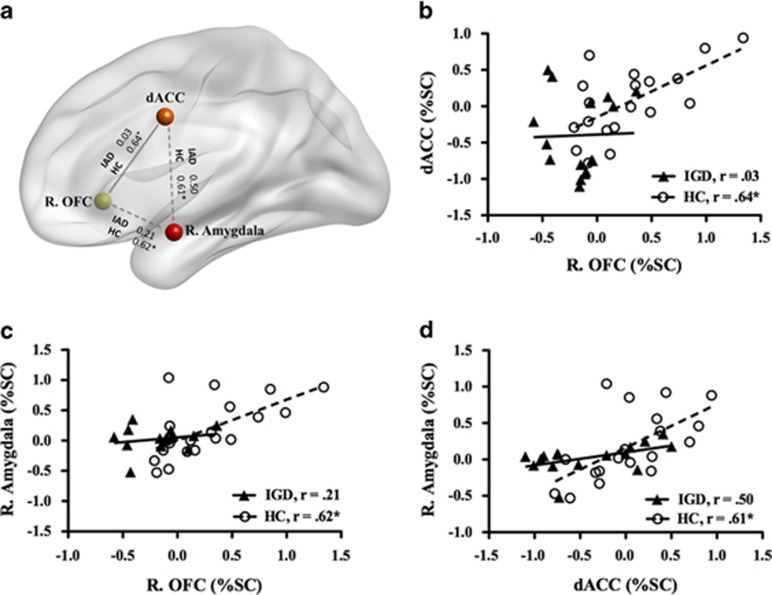
Correlation among the regions of interest (ROIs) in adolescents with Internet gaming disorder (IGD) and healthy control (HC). (**a**) Correlation results in each group. (**b**) Correlation between dorsal anterior cingulate cortex (dACC) and right orbitofrontal cortex (OFC) in the swearing-neutral (SWEA) condition. (**c**) Correlation between the right amygdala and right OFC in the SWEA condition. (**d**) Correlation between the right amygdala and dACC in the SWEA condition. IAD, Internet addiction disorder.

As shown in Figure 2, under the SWEA condition, activation in the right OFC was positively correlated with the dACC (*r*=0.64, *P*=0.006) and right amygdala (*r*=0.62, *P*=0.006) in HC. In addition, activation in the dACC was positively correlated with the right amygdala (*r*=0.607, *P*=0.008) in HC; however, there was no significant correlation in adolescents with IGD. When the IGD group effect was considered as a moderator variable, it revealed that the effect of the dACC (Δ*R*^2^=0.112, ΔF_1,31_=7.08, *P*=0.012, *b*=−0.547, *t*_31_=−2.66, *P*=0.012) on the right OFC decreased more in the adolescents with IGA than in HC.

**Figure 3:**
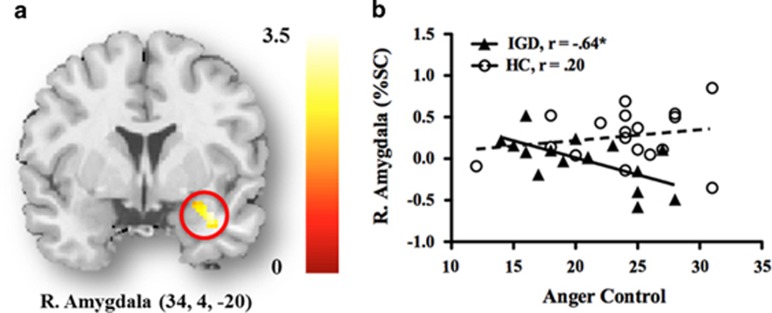
Correlation between activation of the right amygdala and the score on the Anger Control subscale of the STAXI-K in adolescents with Internet gaming disorder (IGD) and healthy control (HC). (**a**) Right amygdala (*x*, *y*, *z*=34, 4, −20). (**b**) Correlation between activities of right amygdala and STAXI-K score in each group.

As shown in Figure 3, the Anger Control subscale of the STAXI-K in adolescents with IGD was negatively correlated with activity in the right amygdala (*r*=−0.64, *P*=0.008) in the SWEA–NEGA condition; this correlation was not significant in HC. The moderating effect for the group revealed that adolescents with IGD showed a negative relationship between right amygdala activity and the score of the Anger Control subscale in the SWEA–NEGA condition (Δ*R*^2^=0.115, ΔF_1,31_= 4.85, *P*=0.035, *b*=−0.412, *t*_31_=−2.20, *P*=0.035).

Because the Beck Depression Inventory and CASS scores were significantly different between both groups, we additionally performed a control analysis by adjusting for the Beck Depression Inventory and CASS scores in the second-level analysis. The results were not remarkably changed.

## Discussion

The study of IGD has increased over the last few years.^47^ Prior studies have reported neuropsychological and neuroimaging research on excessive and addictive use of the Internet^1^ and have noted problems associated with Internet addiction in adolescence.^11, 48, 49^ With the aim of assessing the cognitive control of affective events in IGD, we examined the influence of IGD on neural activity during the processing of swear words in young adolescents.

### Common processing with regard to swear words

Swear words are generally known to induce negative or aggressive feelings.^28^ Swear words involve stronger emotional sensitivity than negative words because the main purpose of swearing is to convey anger, and the display of aggression as its primary meaning is connotative.^50^ Participants of both groups had a faster response to swear words than to negative ones, suggesting that swear words go through more automatic processing compared with negative words. In this study, the activity of the medial OFC in response to swear words may be explained by the involvement of the OFC in the automatic emotion regulation related to reward monitoring.^51^ In a previous study, the activity in the medial OFC was correlated with the monitoring of affective properties^52^ and interaction between arousal and the valence of negative words.^53^

Furthermore, in response to swear words, we found activity in the right superior temporal sulcus, right temporoparietal junction and temporal pole, brain regions known to be involved in social cognition.^54, 55, 56, 57, 58, 59^ This suggests that swear words influence both emotional states and social contexts. Areas related to social interaction such as the right superior temporal sulcus, right temporoparietal junction and temporal pole were involved in social perception while interacting with others.^60, 61^ In addition, the lingual gyrus has been associated with negative stimuli and visual attention.^62^ Therefore, the results of this study indicate that swear words induce strong activity in the brain regions associated with emotional processing, social cognitive emotion and emotional attention.

### Differences between adolescents with IGD and HC in response to swear words

In a statistical map of group differences, adolescents with IGD showed less activation in regions related to language and emotional processing, such as the left inferior frontal gyrus and caudate nucleus, compared with HC. These differences in activation occurred in the absence of behavioral differences between groups, indicating that the activation pattern of the brain can be shown by IGD without differences in the behavioral response. In previous studies, the left inferior frontal gyrus (BA 44 and 46) was related to semantic processing^63, 64^ and cognitive reappraisal.^62^ The lesser activation of the caudate nucleus in adolescents with IGD compared with HC also confirms automatic processing of swear words in the brain, which is in-line with a previous study that examined self-generated emotion in the caudate nucleus.^65^ Therefore, group differences in the SWEA condition suggest that adolescents with IGD exhibit cognitive and emotional deficits in neural activity. These findings agree with other studies in which individuals with IGD continue playing games even when directly confronted with related negative consequences.^66^

### Altered neural responses in the frontolimbic system to swear words in adolescents with IGD

In this study, the frontolimbic regions, including the lateral OFC, dACC and bilateral amygdala, were considered as ROIs in an investigation of the differences between adolescents with IGD and HC in their reactions to swear and negative words. It has been known that the ventral system, including the amygdala and ventrolateral prefrontal cortex, are associated with strong emotional processing.^67^

HC showed stronger activity in response to swear words in the dACC and right OFC compared with adolescents with IGD. They also exhibited significant differences between swearing and negative words in the right amygdala, dACC and right OFC compared with IGD. These activation findings were consistent with regional correlation results. When the IGD group effect was considered as a moderator variable, adolescents with IGD showed lower correlations between the right OFC and dACC and between the right OFC and left amygdala compared with HC.

In this study, the findings suggest that the right lateral OFC is related to cognitive control in response to swear stimuli. The activity in the right OFC is engaged in arousal from negative words^53^ and is correlated with reduced negative emotional experience during emotional regulation.^68^ In particular, the right OFC has a crucial role during implicit emotional regulation.^69^ This finding suggests that HC might reveal emotional sensitivity and cognitive control of presented swear words compared with adolescents with IGD.

The altered correlation between the dACC and lateral OFC shown in adolescents with IGD is a neurobiological marker that is similar to that observed in obsessive-compulsive disorder, which shares compulsive and uncontrolled behavioral tendencies.^18, 19^ One of the IGD diagnostic criteria is the compulsive and persistent use of online games, even when an individual has to stop using it.^6, 7, 8, 9^ In one previous study related to social interaction, the dACC was activated in response to unexpected pain caused by social exclusion, in which individuals were prevented from joining others in social activity.^70^ Therefore, increased activation of the dACC in HC toward the SWEA condition implies a neural response related to the pain of social rejection resulting from being excluded from an important social relationship. On the other hand, the deactivation of the dACC related to social pain induced by feelings resulting from social rejection^71^ suggests that adolescents with IGD might reveal a flat affect in social emotional processing. In previous studies, the dACC contributed not only to pain related to social rejection^72, 73, 74^ but also to cognitive control^75^ and conflict monitoring.^76, 77^ Therefore, these findings suggest that HC processed swear words through emotional regulation and cognitive monitoring. Considering the role of the dACC in error monitoring, cognitive control and conflict management,^14^ these observations indicate that the cognitive control may fail to intervene in the processing of emotionally provoking words in adolescents with IGD.

These differences in regional correlations between adolescents with IGD and HC could be attributed to altered brain structure in adolescents with IGD. Structural imaging studies reported that adolescents with IAD had significantly lower white matter integrity, as measured by fractional anisotropy, than HC in the orbitofrontal white matter and cingulum^16^ and lower brain gray matter density in the ACC.^15^ Therefore, the alterations of the functional correlation between the dACC and OFC, observed in our current study, may be associated with IGD, even though the causal interpretation should be cautious.

The amygdala also has a major role in emotional processes^78^ and the neural response, and increased activation in the amygdala and decreased activation in the OFC are observed in response to social threats in individuals with impulsive aggression.^79^ Bechara *et al.*^80^ suggest that the amygdala and the OFC are involved in emotional processing; however, emotion modulates memory in the amygdala and decision-making in the OFC. Rats with an intact OFC and amygdala lesions failed to learn appropriate stimulus–outcome association and perform goal-directed behaviors.^81^

In this study, adolescents with IGD reported withdrawal, distress and problems of academic functioning caused by overuse of Internet gaming. Therefore, adolescents with IGD experiencing difficulties in control over Internet gaming might have cognitive deficits associated with the adjustment of negative emotion compared with HC.

### Negative correlation between the amygdala and anger control in adolescents with IGD

The current study found that in adolescents with IGD, the score on the Anger Control subscale of the STAXI-K was negatively correlated with the activity in the right amygdala. The Anger Control subscale was used to measure the ability of an individual to control angry feelings.^42^ This result indicates the important role of the amygdala in the control of aggression in adolescents with IGD. In other words, adolescents with IGD who showed higher activity in the right amygdala reported a lower ability to control anger toward swear words compared with HC. In previous studies, exposure to strong language and verbal offenses on the Internet increased adolescent verbal aggression,^82^ and those who played Massively Multiplayer Online Role-Playing games and were identified as ‘problematic players' scored higher on verbal aggression.^83^ In particular, adolescents with Internet addiction were more likely to exhibit aggressive behaviors, and this association was more significant among adolescents in junior high school than those in senior high school.^20^

In summary, the present study provides specific evidence of alterations in emotional processing between adolescents with IGD and HC. Although there were no group differences in the behavioral responses, adolescents with IGD compared with HC exhibited reduced activation in the dACC, a brain region related to social rejection, and the right OFC, a brain region related to emotional regulation, during the swear word condition. These findings suggest that neuronal responses in adolescents with IGD compared with HC reflect a deficit in the controlled processing of swear words. In addition, the adolescents with IGD showed different regional correlations in the frontolimbic regions during the swear word condition, and, in particular, the functional activation of the amygdala was negatively related to anger control in adolescents with IGD. These results indicate an important role of the amygdala in the control of aggression in adolescents with Internet addiction. These findings enhance our understanding of social–emotional perception in adolescents with IGD.

### Limitations

The findings in this study are subject to at least four limitations. First, this study did not consider word frequency across conditions and thus could not control the effect of word frequency on behavioral and neural responses. Second, the positive aspect of swearing related to the tolerance of pain, group solidarity and funny words was not considered. We are interested in the influence of the IGD on neural activities during the processing of swear words. Even though the swear word stimuli have not been used in any other sample before, we believe that studying the effect of Internet gaming on the ability for cognitive control in the face of unpleasant stimuli is meaningful because a cyber-violent behavior that many Korean adolescents who play Internet games reported experiencing is swearing. Third, although adolescents with IGD reported psychological and academic problems caused by Internet gaming through the K-scale, the current study was unable to analyze objective variables related to Internet gaming such as logon duration and money spent on the game itself. Lastly, although we controlled for comorbidity such as Attention Deficit/Hyperactivity Disorder and depression through clinical interviews and diagnostic criteria, the various psychological and environmental variables of participants could not be considered as factors. It is suggested that the association of these factors should be investigated in future studies.

## Figures and Tables

**Table 1:** Demographic characteristics of the adolescents with IAD and HC

	*IAD (*n=*16)*	*HC (*n=*19)*	P*-value*
Age (years)	13.63±1.03	13.37±0.90	0.435
BDI^a^	12.53±11.90	5.25±4.99	0.032*
CASS^b^	32.25±15.50	14.38±8.65	0.001**
K-WISC: block design	10.44±3.14	10.84±3.28	0.713
K-WISC: vocabulary	8.63±2.63	10.37±3.22	0.092
K-Scale	37.56±5.16	24.68±4.45	0.000**
STAXI-K Anger Control	20.63±4.47	23.74±4.69	0.054
Time for Internet excluding Internet game use per week (h)	6.75±5.58	8.53±4.93	0.324
Time for Internet game use per week (h)	19.44±11.55	7.63±5.44	0.001**
			
*Family monthly income (%)^c^*			*0.390*
Less than $1000	8.3	6.2	
$1000–1999	25.0	25.0	
$2000–2999	25.0	0	
$3000–3999	8.3	6.2	
$4000–4999	16.7	25.0	
$5000–5999	16.7	12.5	
$6000–6999	0	12.5	
$7000–7999	0	12.5	

Abbreviations: BDI, Beck Depression Inventory; CASS, Conners–Wells' Adolescent Self-Report Scale; HC, healthy control; IAD, Internet addiction disorder; K-Scale, Korean Internet Addiction Proneness Scale; K-WISC, Korean Wechsler Intelligence Scale for Children; STAXI-K, Korean version of the State-Trait Anger Expression Inventory.

*P<0.05. **P<0.01.

a One adolescent with IAD and three HC data were missing.

b Three HC data were missing.

c Four adolescents with IAD and three HC data were missing. Group differences were tested using the *χ*^2^-test.

**Table 2:** Behavioral data

	*IAD (*n=*16)*	*HC (*n=*19)*	P-*value*
*Negativity*			
Swearing	2.08±0.44	2.18±0.27	0.425
Negative	2.14±0.49	2.28±0.30	0.329
Neutral	1.35±0.42	1.12±0.24	0.059
			
*Reaction time (ms)*			
Swearing	946.70±114.94	924.11±120.95	0.577
Negative	1036.86±126.37	1123.72±124.49	0.049*
Neutral	973.71±148.41	954.37±142.93	0.698

Abbreviations: HC, healthy control; IAD, Internet addiction disorder.

**Table 3:** Brain regions showing significant activation

*Region*	*Coordinates*			Z*-score*	*No. of voxels*
	x	y	z		
*Regional differences revealed by condition*					
* SWEA>NEGA*					
* *B. Lingual gyrus	−14	−78	−6	5.23	9107
	12	−76	0	4.84	1301
* *R. Superior temporal sulcus	56	−16	−8	4.41	694
* *R. Postcentral gyrus	64	2	16	3.97	60
* *B. Orbitofrontal gyrus	0	34	−14	3.86	262
	2	34	−12	3.74	181
* *R. Temporal pole	36	6	−18	3.72	78
* *R. Temporoparietal junction	64	−40	18	3.66	239
* *L. Precuneus	–8	−50	48	3.18	55
* *R. Rolandic operculum	48	−10	16	3.08	60
* SWEA<NEGA*					
* *None					
					
*Group differences in SWEA*					
* IAD<HC*					
* *L. Inferior frontal gyrus	−36	42	2	4.96	1383
* *L. Caudate nucleus	−4	18	4	4.08	71
* *R. Middle temporal gyrus	66	−42	−2	4.03	83
					
*Group differences in NEGA*					
* IAD>HC*					
* *R. Superior temporal gyrus	58	−24	12	5.83	313

Abbreviations: B, bilateral; FDR, false discovery rate; HC, healthy control; IAD, Internet addiction disorder; L, left; NEGA, negative condition; R, right; SWEA, swearing condition.

Clusters with peak-level FDR-corrected *P*<0.05 and more than 50 voxels are reported.
